# Perspectives of Insulating Biodegradable Composites Derived from Agricultural Lignocellulosic Biomass and Fungal Mycelium: A Comprehensive Study of Thermal Conductivity and Density Characteristics

**DOI:** 10.3390/biomimetics9110707

**Published:** 2024-11-18

**Authors:** Maryna Babenko, Yevhen Kononets, Petr Bartos, Ulrich Pont, Frantisek Spalek, Tomas Zoubek, Pavel Kriz

**Affiliations:** 1 Faculty of Civil Engineering, Slovak University of Technology in Bratislava, Radlinského 2766/11, 810 05 Bratislava, Slovakia; 2 Faculty of Forestry and Wood Technology, Mendel University in Brno, Zemědělská 3, 613 00 Brno, Czech Republic; 3 Faculty of Agriculture and Technology, University of South Bohemia in Ceske Budejovice, Studentska 1668, 370 05 Ceske Budejovice, Czech Republic; 4 Faculty of Education, University of South Bohemia in Ceske Budejovice, Jeronýmova 10, 371 15 Ceske Budejovice, Czech Republic; 5 Faculty of Architecture and Planning, TU Vienna, Karlsplatz 13, 1040 Vienna, Austria

**Keywords:** biocomposites, agricultural biomass, mycelium, thermal characteristics, density characteristics, insulation materials, lignocellulosic materials

## Abstract

The research suggests a production method of insulating composites created from lignocellulosic agricultural biomass with fungal mycelium as a binder agent and offers a deeper investigation of their thermophysical properties. Particularly, the samples were meticulously evaluated for density and thermal conductivity. The function was built on the suggestion by the authors regarding the thermal conductivity-weight ratio indicator. The metric was initially introduced to assess the correlation between these parameters and was also applied to qualitatively evaluate the biocomposite among other commonly used natural insulations. An applied polynomial trend analysis indicated that the most effective densities for the wheat, hemp, and flax, which were 60, 85, and 105 kg·m^−3^ respectively. It was determined that the optimal density for wheat and hemp composites corresponded to values of 0.28 and 0.20 W^−1^·kg^−1^·m^4^·K of the coefficient, respectively. These values were superior to those revealed in other common natural insulating materials, such as cork, cotton stalks, hempcrete, timber, etc. As a result, the proposed insulating material may offer numerous opportunities for application in industrial settings of civil engineering.

## 1. Introduction

The thermal conductivity (TC) of insulation materials is the most important factor for energy efficiency. Thermal conductivity, denoted by the symbol *λ*, is a fundamental characteristic of a material. It quantifies the rate of heat transfer in watts (*W*) across a 1 m² area and 1 m thickness of the material when there is a temperature difference of 1 Kelvin (*K*) between the two surfaces along the direction of heat transfer. The standard unit for thermal conductivity is W·m^−1^·K^−1^. The thermal conductivity at a mean temperature of 10 °C is considered as determined in accordance with the principles of the method described in various standards such as EN 12664, EN 12667, EN 12939, and ASTM C518. A material is considered a thermal insulator if its conductivity is below 0.07 W·m^−1^·K^−1^ (0.06 kcal·m^−1^·h^−1^·°C^−1^) [1,2,3]. It determines how well they can prevent heat loss or gain in a building, which directly affects energy consumption. Using materials with lower thermal conductivity can create a comfortable indoor environment and reduce the need for heating, cooling, and ventilation systems, leading to substantial energy savings and environmental benefits [4,5]. Similarly, the decreased weight of insulation materials has the potential to greatly reduce both the energy consumption and expenses associated with transportation in construction. Especially, it may hold higher significance for various other applications, such as transportation containers, household appliances, and aircraft [6].

Mycelium is a type of fibrous fungus and presents a distinctive and groundbreaking method for low-energy production and waste control. Materials derived from mycelium are cost-effective, lightweight, capable of decomposing naturally, and have a minimal ecological footprint [7]. Girometta et al. [8] described efficient transformation of agricultural biomass, usually as an agricultural waste, into sustainable substitutes for insulating materials, offering an energy-efficient route for organic synthesis. Some investigations found that the feedstock used in mycelium-based composite materials affects their mechanical and thermal properties [9,10,11]. Flax, hemp, and wheat straw containing fillers are preferred due to their relatively low moisture content of 7–13%, density of 60–134 kg·m^−3^, and strength at 10% deformation of 0.36–0.38 MPa [12,13]. One research fabricated composite mycelial specimens with densities even falling within the range of 30–50 g·cm^−3^ [14], while composites incorporating forestry by-product substrates, such as sawdust, exhibit elevated densities ranging between 87 and 300 kg·m^−3^ [15]. The thermal conductivities established for these composites are as follows: 0.058 W·m^−1^·K^−1^ for mycelium-flax composite, 0.040 W·m^−1^·K^−1^ for mycelium-hemp composite, and 0.042 W·m^−1^·K^−1^ for mycelium-wheat composite within a density range of 94–135 g·cm^−3^ [12]. In general, mycelium composites exhibit a low thermal conductivity ranging from 0.05 to 0.10 W·m^−1^·K^−1^, which is comparable to that of conventional insulation materials [16,17] and potentially could even replace them [18,19].

There are other promising insulation materials fulfilling most of the requirements. However, no single material is high performing in every aspect and therefore the choice of insulating material must take into account given conditions of the specific structure. Common thermal insulation materials in construction include mineral fiber, expanded polystyrene (EPS), extruded polystyrene (XPS), and polyurethane rigid foam (PUR). They provide good thermal conductivity (0.030 to 0.040 W·m^−1^·K^−1^) and are easy to handle, but they are non-renewable, have high embodied carbon, and can be toxic when burned [5]. These materials have significant environmental impacts due to their high embodied carbon content. For example, cellulose fiber (polysaccharide (C_6_H_10_O_5_)_n_) from recycled paper or wood pulp is renewable and recyclable. Table 1 illustrates the main components of lignocellulosic biomass, such as wheat, flax, and hemp.

Depending on the moisture level, cellulose insulation’s usual thermal conductivity ranges between 0.040 W·m^−1^·K^−1^ and 0.050 W·m^−1^·K^−1^ [5]. Another research [26] investigated waste wood fibers as a thermal insulating material in a timber frame wall construction and established the thermal conductivity in the range within 0.048 and 0.055 W·m^−1^·K^−1^. Cotton stalks are one more renewable and bio-based insulating material. Using high frequency hot pressing, ref. [27] created a cotton stalk fiberboard without any chemical additions. The thermal conductivity of the fiberboards ranged from 0.0585 to 0.0815 W·m^−1^·K^−1^. Thermal insulating cork, which primarily manufactured from cork oak, is also utilized in isolating. Compared to fiber materials, it has the benefit of having a very high compressive strength. Cork has a thermal conductivity between 0.040 and 0.050 W·m^−1^·K^−1^ [5]. A promising bio-based contender for use as a thermal insulating material is hemp stem fiber. This material has a few more benefits, including the ability to ward off rodents, and great durability and other potentials [28,29]. For example, one investigation [28] defined the thermal conductivity of 0.052 W·m^−1^·K^−1^ for hemp stem fiber samples with the phenol formaldehyde (PF) resin glue at density of 300 kg·m^−3^. Another research [29] demonstrated *λ*-value on the close meaning of 0.051 W·m^−1^·K^−1^ for a hemp biomass with only gypsum binder at the slightly higher density of 332 kg·m^−3^. One more potential eco-friendly insulating material is flax straw. It is also renewable, biodegradable, and may be recycled or composted at the end of its useful life because it is manufactured from the waste products of the flax plant [30]. For example, Cerny et al. (2023) investigated the thermal performance of flax straw separately and combined with a liquid glass Na_2_O(SiO_2_) as a binder [31]. The material’s thermal conductivity was discovered in the minimum value of 0.072 W·m^−1^·K^−1^, and the binder had little effect on the material’s thermal conductivity significantly increasing its fire resistance. Hempcrete (a combination of hemp shavings and lime binder) is a lightweight material that offers an excellent thermal conductivity property that is measured between 0.087 and 0.100 W·m^−1^·K^−1^ at 300–400 kg·m^−3^ [32,33]. Vacuum insulation panels (VIPs) are an alternative super-insulator with a thermal conductivity of 0.004 W·m^−1^·K^−1^, but they degrade over time. They are more expensive than traditional insulators and can lose effectiveness if punctured [34].

A similar biocomposite, for example, obtained from various substrate materials, including wood pulp, millet grain, wheat bran, natural fiber, and calcium sulfate with the addition of *Irpex lacteus* mycelium under three different preparation protocols, showed the best average thermal conductivity of 0.06 W·m^−1^·K^−1^ (0.05–0.07 W·m^−1^·K^−1^) and the average density of 180 kg·m^−3^ (165–195 kg·m^−3^) [35]. Another work established the thermal conductivity for chopped hemp, flax and wheat straw fibers combined with a white rot mycelium spawn *Trametes versicolor*, and the samples showed the best values for flax biomass of 0.057 W·m^−1^·K^−1^, for hemp and wheat, respectively, and 0.040 W·m^−1^·K^−1^ and 0.041 W·m^−1^·K^−1^ at densities of biocomposites of 134, 99 and 94 kg·m^−3^ relatively [12]. Other research investigated two more composites derived from cellulose fiber and rapeseed bagasse with grown *Ganoderma lucidum* on the substrates, where the first composition demonstrated the best thermal conductivity of 0.085 W·m^−1^·K^−1^ with the average density of 373 kg·m^−3^, while the second one had the λ-value of 0.057 W·m^−1^·K^−1^ and the *ρ*-value of 156 kg·m^−3^ [36]. One study employed the fungal species Ganoderma williamsianum and Lentinus sajor-caju to create mycelium-based composites, which demonstrated superior physical properties including high density of from 221.05 kg·m^−3^ for rice straw and up to 340.31 kg·m^−3^ for wooden-based sawdust, low water absorption, and minimal shrinkage, as well as enhanced mechanical properties such as high compression, tensile, flexural, and impact strengths compared to other species examined [37]. However, it is important to note that in the context of insulating materials, the increased density may be considered a disadvantage.

Hence, there exists a variety of insulation materials including similar composites derived from agricultural biomass and mycelium. However, not all are environmentally sustainable and possess competitive physical and energy-efficient parameters. Moreover, there is a lack of evidenced techniques for comparing insulating materials using a combined indicator that considers weight and thermal insulation characteristics. Current research suggests the production technology and optimal formulation of a promising biocomposite derived from agricultural lignocellulosic biomass with mycelium as a binder. Earlier, sufficient achievements regarding analogous biomass-mycelium composites have been previously demonstrated through a range of literature that has gradually emerged since the influential study conducted by Greg Holts and colleagues in 2012 [38]. Some others, for example [39,40], reported good acoustic performance of this type of mycelium in insulating materials, others [36,41,42] demonstrated rapid colonization of the substrate, good thermal properties with the lowest density and good dimension stability of materials [38], in addition to proving good fire-resistant capacity by [40,43].

Current investigation also makes an in-depth analysis of the thermal conductivity and weight characteristics of the suggested biocomposites, comparing them with other insulating choices through the application of a alternative ratio coefficient.

The hypothesis of this investigation is that “a biocomposite based on agricultural lignocellulosic biomass and mycelium spores has the best weight-thermal conductivity ratio among known biodegradable insulators”. The primary objective of this research is to incorporate fungal mycelium as a binding agent to develop and characterize samples of insulating materials using three different types of lignocellulosic agricultural biomass: hemp, flax, and wheat straw. The secondary objective is to present a new qualitative Thermal Conductivity-Weight Ratio (TC-WR) coefficient for evaluating insulation alternatives and to quantitatively compare them by thermal conductivity and density characteristics. The third objective is to project optimal production meanings of density (O) for each basis of lignocellulosic biomass at the max value of TC-WR value.

## 2. Materials and Methods

### 2.1. Sample Formation Technique

First, the study took a closer look at three lignocellulosic ‘agricultural waste’ in particular at wheat, hemp and flax basis, with making by five equal samples of each type. This amount was determined under resource constraints with an average deviation of the results from the average values for thermal conductivity of only ±0.9%, and for density ±1% (Table 1).

The samples were produced as follows. First, all types of straw were chopped manually into fractions (particle size) of 5–15 mm and moistened. Next, the material was sterilized in a constant climate chamber (KBF 240, Binder GmbH, Tuttlingen, Germany) at 100 °C and 90% relative air humidity for 3 h. The straw mass was then inoculated with Ganoderma lucidum mycelium (Reishi fungi). Already prepared commercial fungi mycelium pack was used in the experiment test, which can be acquired in available marketplaces such as Alibaba.com.

**Figure 1:**
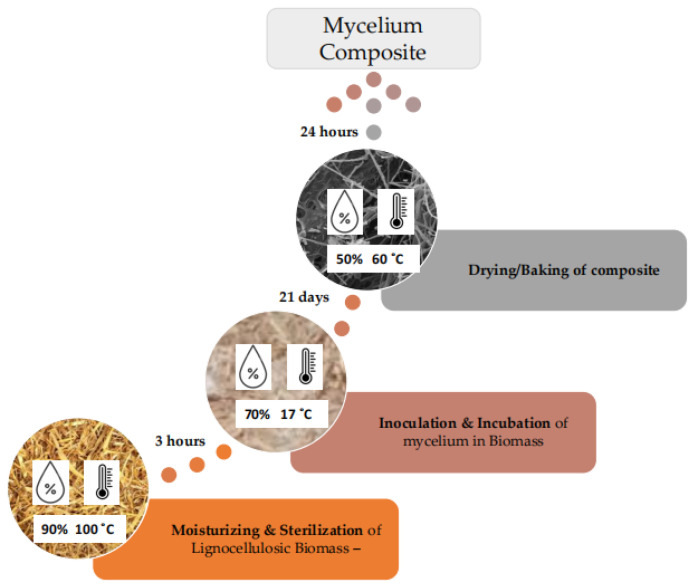
Methodology of sample formation technique.

**Figure 2:**
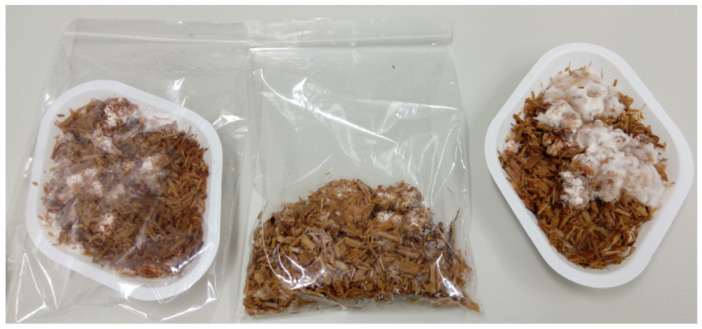
First stage: the process of fungi mycelium growth in a small substrate volume.

**Figure 3:**
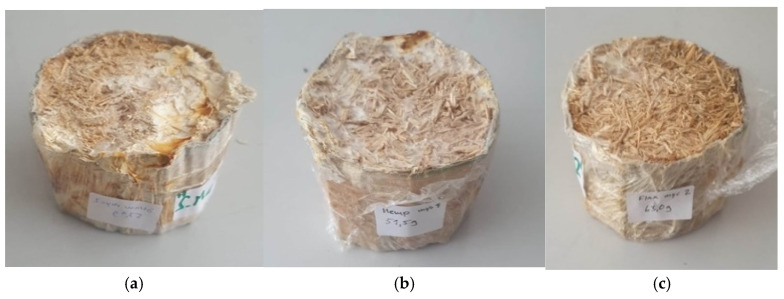
(**a**) Chopped wheat with mycelium; (**b**) Chopped hemp with mycelium; (**c**) Chopped flax with mycelium. Source: Made by the authors.

**Figure 4:**
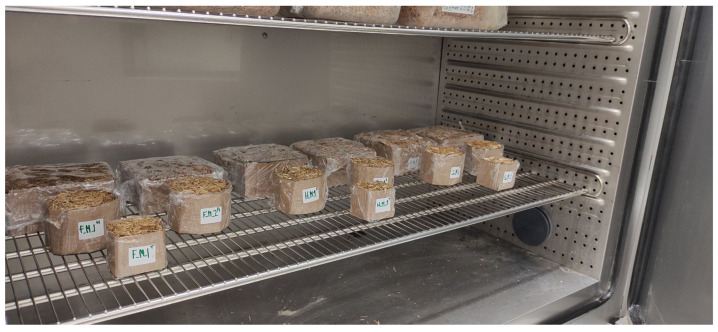
Preparing samples of each type in a climate chamber: wheat, hemp, and flax straw.

In practice, the mycelium growth process was established empirically by the authors (Figure 1) and the growth process itself was conducted in two main stages: (1) in a pile of straw for one week (growing fungi mycelium for 7 days in a small volume bulk substrate in a polyethylene bag with ventilated holes) (Figure 2) and (2) manually pressed directly into prepared cylindrical forms (Figure 3). The forms are removable cardboard, wrapped in polyethylene with ventilation holes for two weeks (14 days), totaling 21 days. Preliminary, the cardboard was made from a cellulose multilayer material, providing vapor and air permeability with a thickness of 3 mm and a density of 1890 g·m^−2^ and rolled them into necessary cylinders. Both are under similar environmental conditions in a climate chamber at 16–17 °C and 70–80% relative air humidity. After the growth process, the samples were removed from their forms and baked (dried) for 24 h (1 day) in a climate chamber at 60 °C and 50% relative air humidity. Thus, 15 samples had mycelium as a binder with different pure substrate fillers of wheat, hemp, and flax chopped straw (by 5 for each type) *(*Figure 3a–c, resp.; Figure 4). Although the mycelium growth in all 15 samples occurred under identical conditions, the resulting internal geometry of mycelium in straw, perimeter size, and density was slightly different. The samples created using cylindrical form with the diameter 96 mm and height of 65 mm.

### 2.2. Thermal Conductivity and Density Measurements

The transient method, also known as the non-steady method, was employed in accordance with ASTM D5334-08 [X+1] utilizing a thermal needle probe. Portable heat transfer analyzer (Isomet 2114, Applied Precision s.r.o., Bratislava, Slovakia) was used for measuring the thermal conductivity *λ* [W·m^−1^·K^−1^] of the samples. We performed the experiment in environmentally controlled laboratory area on the Faculty of Civil Engineering of the Slovak Technical University in Bratislava at constant 20 °C and 50% relative humidity to ensure stability throughout the measurement process. The Isomet 1224 is designed for high-precision thermal conductivity measurements and is equipped to compensate for minor ambient variations. In addition, the polyethylene cover was used to isolate the straw-mycelium composite samples while TC measuring (cca. 2 h), minimizing heat exchange with the surrounding environment, especially through convective air currents. In general, the heat transfer analyzer is a device that can assess heat transfer properties in various isotropic materials like cellular insulating materials, plastics, glasses, and minerals. The heat transfer analyzer has an accuracy of 5% ± 0.001 W·m^−1^·K^−1^ for thermal conductivity measurements between 0.015 and 0.70 W·m^−1^·K^−1^, and 10% for measurements between 0.70 and 6.0 W·m^−1^·K^−1^. The measurement reproducibility is 3% ± 0.001 W·m^−1^·K^−1^. These performance characteristics were within the acceptable statistical range and do not significantly affect the research findings [44].

**Figure 5:**
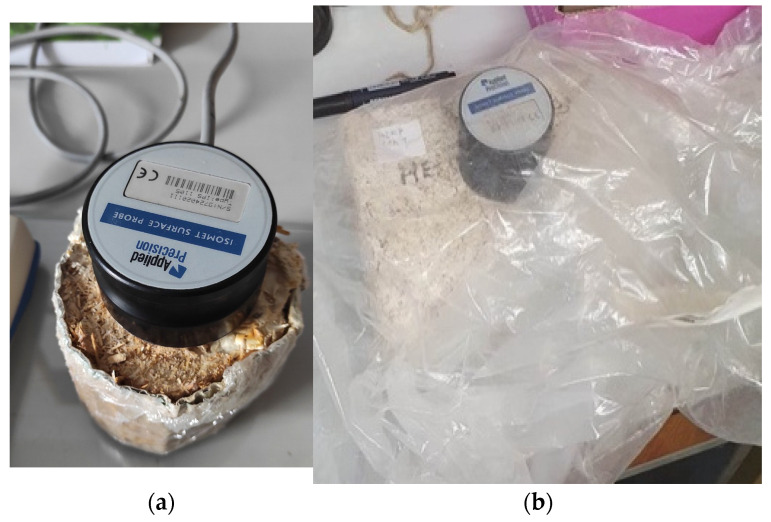
Measuring the thermal conductivity of the samples (**a**) the surface probe; (**b**) plastic foil covering for the samples.

In order to operate, the heat transfer analyzer had to be connected to the surface probe for hard materials, and the measuring range was set to 0.04–0.3 W·m^−1^·K^−1^. Each formed sample was measured by two measurements from two tangential surfaces (adjacent faces), and the median value was recorded between the two as *λ* [W·m^−1^·K^−1^] (Table 1). When the device was properly set up, the samples were prepared by means of measuring the dimensions and weight, and they were placed on a glass board. The surface probe was then positioned on top of the sample (Figure 3a) and a plastic foil was applied to cover the sample and probe (Figure 5b) in order to ensure the environmental conditions to stay the same for the duration of the measurement. The sample was then tested for duration of approximately five hours, after which the result was recorded.

**Figure 6:**
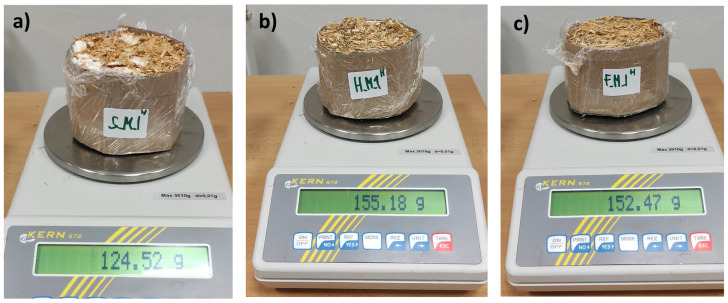
Representative samples of wheat-based (**a**), hemp-based (**b**) and flax-based (**c**) composites injected with mycelium during the process of forming and weighing samples before drying.

**Figure 7:**
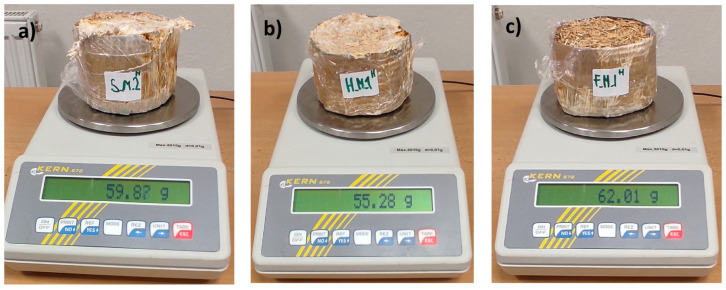
Representative samples of wheat-based (**a**), hemp-based (**b**) and flax-based (**c**) composites binded with a fungal mycelium after drying.

The density *ρ* [kg·m^−3^] of the material was measured manually for each of the two samples with laboratory scales (Kern 572, KERN & SOHN GmbH, Balingen, Germany) (max. measurement mass *m* = 3010 g; deviations *d* = 0.01 g) (Figure 6 and Figure 7) and a manual measuring ruler. The results of both measurements were recorded within the range of values *ρ*_min_–*ρ*_max_ [kg·m^−3^] obtained for the material. In particular, the density of the composition was measured using the equation [45]:

(1) $$\rho =\frac{m}{\pi \times ({\frac{D}{2})}^{2}\times h}\;\mathrm{for}\;\mathrm{cylindrical}\;\mathrm{samples}$$

where *m* is the mass of the material (in kg); π is 3.14159; *D* is the diameter of the cylinder samples (in meters); and *h* is cylinder height (in meters). Diameter and height were the nominal dimensions of the molds used to produce the samples. The average values of the parameters were calculated as arithmetic means. Moisture content *MC* (%) in samples was manually measured by weight drying at 20 °C following ISO 16979:2003 [46] by formula (Figure 6 and Figure 7):

(2) $$MC\;(\%)=\frac{Initial\;weight\;[kg]-Weight\;after\;drying\;[kg]}{Initial\;weight\;[kg]}\times 100$$

### 2.3. TC-WR Indicator and Polynomial Trend Analysis

A thermal conductivity to weight ratio (TC-WR) measurement in given form was firstly introduced by the authors and was used to qualitatively evaluate the effectiveness in reducing heat transfer relative to its weight. Since the indicator is inverse, it means that the higher the ratio, the more competitive the material is in the list of alternatives. For insulation materials, both factors were taken into account when determining the efficiency using the equation, which was suggested by the authors in current research:

(3) $$\mathrm{Thermal}\;\mathrm{Conductivity}-\mathrm{Weight}\;\mathrm{Ratio}\;\mathrm{coefficient}=\frac{1}{\lambda \times \rho }(W^{-1}\cdot {\mathrm{kg}}^{-1}\cdot m^{4}\cdot K)$$

where *λ* is the thermal conductivity of the material in W·m^−1^·K^−1^, and *ρ* is the density of the material in kg·m^−3^. Higher values indicate better insulation efficiency.

Figure 9 presents the TC-WR curve, which illustrates the TC-WR values and the correlation between samples of each biomass category. To determine the optimal density point (*O*-density) at the peak of the TC-WR polynomial trend line, a mathematical prediction function was employed. A polynomial trend curve is a type of prediction utilized in data analysis and graphing to depict the correlation between independent and dependent variables through a polynomial equation. These trend lines had the flexibility to bend and curve in order to accommodate a wide array of data patterns, making them valuable for capturing intricate relationships [47]. Potentially, it is able to generalize and accurately predict other meanings of TC-WR at wide range of density. The general form of a polynomial equation of degree *n* is [48]:

*y* = *a*_0_ + *a*_1_*^x^*+ *a*_2_*x*^2^ + *a*_3_*x*^3^ +⋯+ *a_n_x^n^* (4)

where *y* is the dependent predictable variable meaning density of the material, and *x* is the independent variable of density range within 0–250 kg·m^−3^. The actual significant range was considered to be between 50 and 250 kg·m^−3^ even if the maximum value will be less than the density of <50 kg·m^−3^ since, according to authors’ observation, such samples will not be dense enough to hold a shape for practical construction purposes. In general, composites that incorporate agricultural by-product fillers, such as bast fibers or straw, generally exhibit lower densities, ranging from 60 to 130 kg·m^−3^, compared to those that utilize forestry by-product materials like sawdust, which can reach densities between 87 and 300 kg·m^−3^ [15]. One research declared densities for treatments between 66.5 and 224 kg·m^−3^ [38]. Thus, *a*_0_, *a*_1_, *a_n_* are the coefficients that play a crucial role in defining the precise form and orientation of the curve. These coefficients as well as the best fit function were described by software (Microsoft Excel, Microsoft Corporation, Redmond, WA, USA) and derived using polynomial regression, a method that computes values to closely match the given data points [49] which applies to five measured samples for each of three types of composite. All five samples of each type of biomass do not differ in composition, but they do differ in density, since the method of creating the forms involved manual compressing.

### 2.4. Scanning Electron Microscope (SEM) Analysis

**Figure 8:**
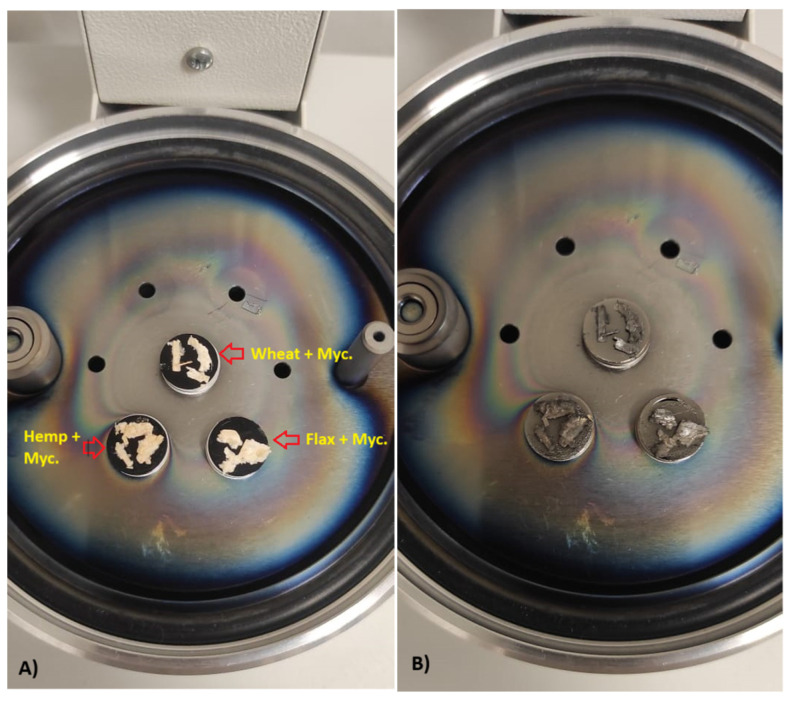
Prepared composites for SEM analysis (**A**): Coated particles with non-oxidising metals (**B**).

Images of the composite surface were captured using a VEGA 3 SBU scanning electron microscope from TESCAN (TESCAN GROUP, a.s., Brno, Czech Republic). The SEM examination focused on three studied types of biomass particles measuring in average 6.5 mm (long) × 1 mm (width), which are pre-installed in rotary pumped sputter coater Quorum SC7620 (Quorum Technologies Limited, Laughton, UK) for coating with non-oxidizing metals like Au, Au/Pd, Ag, Pt and Pt/Pd required for SEM analysis (Figure 8). SEM analysis was conducted within a high vacuum environment, maintaining a pressure of less than 1 × 10^−2^ Pa. A 10 kV electron beam, achieving a resolution of 3.0 nm (as defined by the Vega3 TESCAN) [50], was utilized, with a working distance ranging from 7 to 9 mm. Adjustments to the incident probe current, as well as contrast and brightness settings, were made to ensure high-quality imaging for surface imaging (SE images). Each individual microscope image includes detailed information regarding the microscope mode, detector mode, working distance in millimeters, field of view in millimeters, scale, and magnification level.

## 3. Results

The result of the respective density and thermal conductivity for tested wheat, hemp, and flax -based samples with a mycelium binder are presented in Table 2. All samples were then ranked in descending order by the quality indicator TC-WR among some common insulation materials (Table 3). It can be noted that the different mycelium-based materials show similar thermal conductivity properties in the range of 0.043–0.056 W·m^−1^·K^−1^.

TC-WR coefficient showed quite similar meanings for flax and hemp raw fillers of 0.127 and 0.123, respectively, but the wheat-based sample appeared with a higher value of 0.148 units (Table 3). In turn, the best indication demonstrated non-organic insulations EPS, VIP, PUR, XPS reaching numbers by a wide margin between 0.840 and 1.389 units because of the high-performed average TC, which was between 0.020 and 0.040 W·m^−1^·K^−1^ (except VIP) and extra light density with the average range of 15–40 kg·m^−3^ [51,52,53,54,55,56]. VIP is quite dense (200 kg·m^−3^), but it has an extremely low thermal conductivity of 0.004 W·m^−1^·K^−1^ [6,57], which allowed it to become the first among the observed insulators in terms of qualitative ‘TC-Weight’ indicator.

Among the bio-based materials, the best values of TC-WR coefficient were revealed by sheep wool, cellulose fibers and mineral wool, respectively (Table 3). Herein, the quality ‘TC-weight ratio’ indicator of samples with mycelium fell below the leading samples from the organic matter because of a bit higher TC and much higher density in compare with sheep wool and cellulose fibers insulations. At the same time, the widespread mineral wool has quite similar density, though it has slightly better confirmed TC values between 0.034 and 0.045 [58,59].

Pure organic materials like cotton stalks and light earth, as well as bio-based composite materials such as hempcrete and flax-glass, showed TC-WR coefficient ratios between 0.041 and 0.057 and were lower in the ‘rank’ (Table 3) than the ‘agriwaste-mycelium’ composites studied. Cork showed slightly a lower meaning of 0.139 than wheat and mycelium composite (0.148) but higher than other studied composites of lignocellulosic biomass. Pure softwood showed the worst thermal conductivity to weight ratio coefficient, which is 0.015.

**Figure 9:**
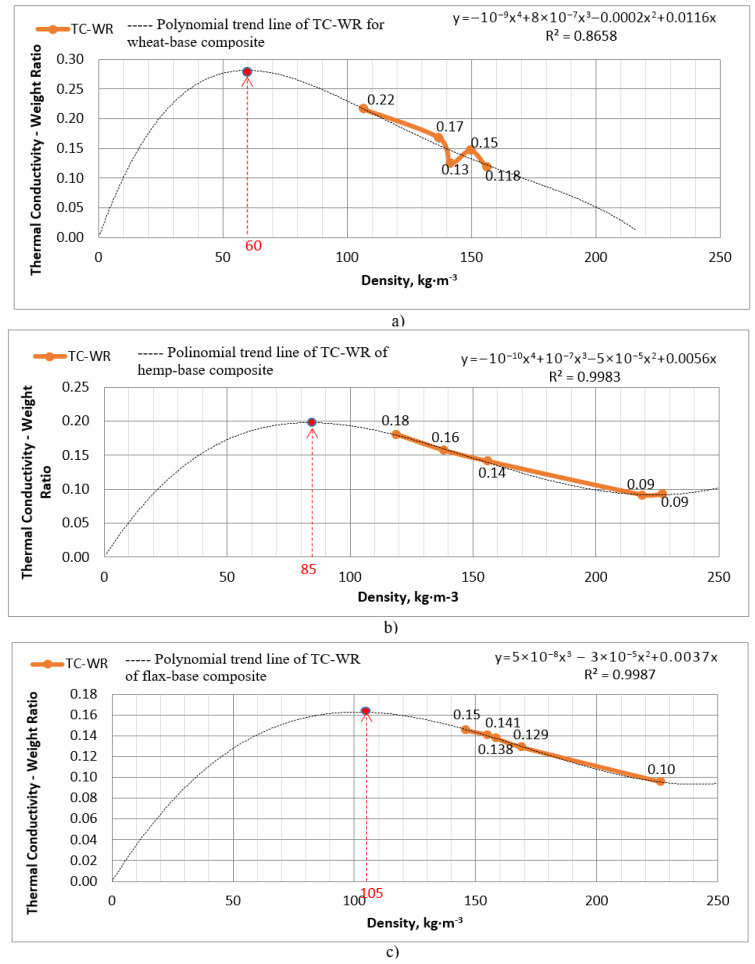
TC-WR values for measured types of biomass samples (orange line) and polynomial trend lines of TC-WR for density range of 0–250 (kg·m^−3^) with the approximation reliability values (*R*^2^) between 0.865 and 0.998 and *O*-density point for wheat (**a**), hemp (**b**) and flax (**c**) straw composites with mycelium binder.

In Figure 9, there are polynomial trend lines of TC-WR for three types of lignocellulosic agricultural biomass. In particular, the prediction curve for wheat-based composite has the lowest approximation reliability value (*R^2^*), namely 0.865, which is still high enough to explain the ratio. *R*^2^ is a statistical measure that indicates the proportion of the variance in the dependent variable that is predictable from the independent variables in a regression model. The other two curves with hemp- and flax-based materials have the highest values of *R*^2^ which are 0.998 and 0.998, respectively, and they indicate a better fit of the regression model to the data, suggesting that the independent variables are more effective at explaining the variability in the dependent variable. The range of significance lies between 50 and 250 kg·m^−3^. Notably, the maximum values of TC-WR curves for wheat-, hemp-, and flax-based composites exceed a density value of 50 kg·m^−3^ in all three cases (Figure 9a–c) and specifically reaching their max point at density meanings of 60 kg·m^−3^, 85 kg·m^−3^ and 105 kg·m^−3^, respectively.

**Table 3:** Aggregated findings of different basic insulation materials are sorted by ‘Thermal Conductivity—Weight Ratio’ in descending order.

Insulation Material	Thermal Conductivity at 10 °C, *λ*_min_–*λ*_max_ [W·m^−1^·K^−1^]	Dry Density (Own Mean.), *ρ*_min_–*ρ*_max_ [kg·m^−3^]	Bio ^1^	Reference	Average Thermal Conductivity *, *λ_Avg_* [W·m^−1^·K^−1^]	Average Density *, *ρ_Avg_* [kg·m^−3^]	Thermal Conductivity-Weight Ratio *, 1/*(λ_Avg_*·*ρ_Avg_*) [W^−1^·kg^−1^·m^4^·K]
Vacuum Insulation panels (VIP)	0.004	160–200	N	[6,57]	0.004	180	1.389
Polyurethane rigid foam (PUR)	0.020–0.030	30–45	N	[51,52]	0.025	38	1.053
Expanded polystyrene (EPS)	0.030–0.040	15–40	N	[53,54]	0.035	27.5	1.039
Sheep wool	0.032–0.054	10–40	Y	[60,61,62]	0.043	25	0.930
Extruded polystyrene (XPS)	0.028–0.040	25–45	N	[55,56]	0.034	35	0.840
Cellulose Fibers	0.036–0.042	40–70	Y	[63,64]	0.039	55	0.466
Wheat + mycelium *Trametes versicolor*	0.042	94	Y	[12]	0.042	94	0.252
Hemp + mycelium *Trametes versicolor*	0.040	99	Y	[12]	0.040	99	0.250
Mineral Wool	0.034–0.045	120–140	Y	[58,59]	0.039	130	0.197
Wheat + mycelium *Ganoderma lucidum* *	0.043–0.056	107–156	Y	Current research	0.048	138	0.148
Cork	0.040–0.050	80–240	Y	[65,66]	0.045	160	0.139
Flax + mycelium *Trametes versicolor*	0.058	135	Y	[12]	0.058	135	0.128
Flax + mycelium *Ganoderma lucidum* *	0.045–0.047	146–220 *	Y	Current research	0.046	171	0.127
Hemp + mycelium *Ganoderma lucidum* *	0.045–0.050	119–227	Y	Current research	0.047	172	0.123
Soybean straw + Mycelium *Ceriporia lacerata*	0.054	160		[67]	0.054	160	0.116
Rapeseed bagasse + mycelium *Ganoderma lucidu*	0.057	156	Y	[36]	0.057	156	0.112
various substrate materials + mycelium *Irpex lacteus*	0.060	180	Y	[35]	0.060	180	0.092
Bark fiber	0.044–0.063	164–276	Y	[68]	0.0535	220	0.084
Flax + waterglass	0.066–0.068	235–284	Y	[31]	0.0672	259.5	0.057
Cotton Stalks	0.058–0.081	150–450	Y	[2,27]	0.070	300	0.048
Hempcrete	0.057–0.066	389–441	Y	[32]	0.0619	365	0.044
Light-earth	0.060–0.120	190–353	Y	[69]	0.090	271.5	0.041
Cellulose fiber + mycelium Ganoderma lucidu	0.085	373		[36]	0.085	373	0.031
Timber (softwood)	0.130	500	Y	[70]	0.130	500	0.015

Legend: *—Established averages by the authors, Bio ^1^—Biodegradability (N—No, Y—Yes).

**Figure 10:**
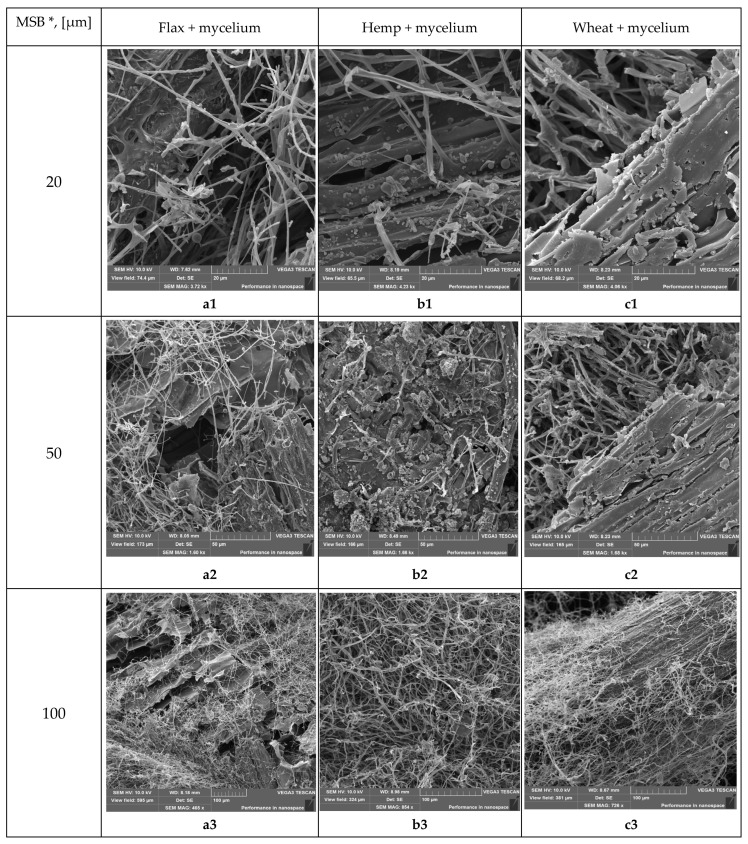

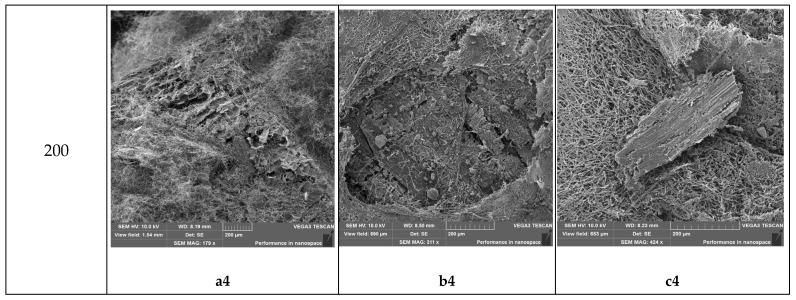
Scanning electron microscopic images of biomass–micelium composites obtained in this study: The surfaces of straw-micelium composites produced from Ganoderma lucidum mycelium with flax straw (**a1**–**a4**), hemp straw (**b1**–**b4**) and with wheat straw (**c1**–**c4**). Legend: MSB *—Magnification scale bar.

The morphological characteristics of straw–micelium composites were examined by SEM. It was found in Figure 10 that, in all three composite types, flax + mycelium (a1–a4), hemp + mycelium (b1–b4), wheat + mycelium (c1–c4), the fungi established robust adhesion to the substrate surfaces, which is a critical factor for material cohesion and structural integrity. The hyphae are intricately interwoven within the composites, creating a dense network that enhances structural integrity and strength. The coverage of the straw surface in all cases was thorough, demonstrating that the fungal growth could adapt to the morphology of this substrate. In particular, it is clearly seen at the 20 μm magnification scale (Figure 10a1,b1,c1). The natural growth behavior of the mycelium played a pivotal role in binding the substrates, demonstrating that fungi not only act as a natural adhesive but also enhance the composite’s integrity by bridging gaps between fibers and ensuring a uniform composite matrix. The morphological characteristics of the lignocellulosic biomass—micelium composites discussed within this study were similar to those in alternative research [10,37,71].

## 4. Discussion

### 4.1. General Perspectives

Despite the painstaking work of measuring thermal conductivity, density, and the qualitative relationship between the first two, other factors also influence the characteristics and quality of insulating materials, they include: durability, resistance to fire, rodents and microorganisms, mechanical strength, toxicity, workability, freezing/thawing cycles, water resistance and cost efficiency, scalability, resource availability, etc. In addition, specific application requirements also play significant roles in selecting the most suitable insulation material, which is out the scope of current research. In summary, our hypothesis was only partially confirmed. We can now state that biocomposites made from agricultural biomass and fungal mycelium demonstrate impressive characteristics regarding weight and thermal conductivity, positioning them among the top bio-based choices available. On the other hand, the evaluation of sheep wool, cellulose fibers, and mineral wool showed a higher coefficient. Although the thermal conductivity values were found to be average in the data examined, we believe that the suggested composites hold the most potential and promise for mass industrial production. Due to its 100% biodegradability, better availability, potentially lower cost of the biocomposite, and lack of regulation compared to other alternatives, it is more attractive than others. Moreover, these materials have the fastest rate of resource renewal, typically within 6 months on average. The mathematical analysis applied indicated a significant potential for reducing the density of suggested these materials while minimizing the decrease in thermal conductivity.

### 4.2. Industrialization and Distinctive Features

In order to facilitate the industrialization of the material and ensure consistent thermal and weight characteristics, it is imperative to maintain strict control over the moisture content, particle size, and pressing force of the samples. Although the article does not delve into these factors in depth, it is worth noting that the samples were exclusively produced for the purpose of the study. Due to some research limitations, mycelium materials, similar to all other materials, are expected to encounter various challenges that may influence their performance. For example, the variation in optimal density among the ostensibly lightest wheat, medium hemp, and heavier flax biomass composites can likely be attributed to the inherent properties of the materials, as the disparity in thermal conductivity is minimal. The study [35] has the same order as wheat-, hemp-, and flax-based with densities of 94 kg·m^−3^, 99 kg·m^−3^ and 135 kg·m^−3^, respectively. In a view of properties of the materials, hemp and flax fibers have very low lignin content (usually under 3%), whereas wheat straw and wood fibers possess high lignin content (higher than 10% and 20%, respectively). Lignin is a polyphenolic thermoplastic compound, similar to that of gluten [72]. Moreover, polyphenolic structures are well known for their strong interactions with proteins [73], which may explain the better adhesive capacity of wheat straw and the formation of stable forms of lower density and greater porosity. In general, the mycelium composites demonstrate a significantly porous microstructure (Figure 10). It is proposed that this porosity plays a crucial role in enhancing thermal insulation while simultaneously decreasing density. The network of interconnected pores is likely to hinder heat transfer, resulting in a reduction in the composite material’s thermal conductivity. Furthermore, the existence of voids contributes to a lower overall material density, rendering these composites lightweight and potentially beneficial for applications that necessitate a decrease in material mass. These results align with the findings of Alaneme et al. (2023) [74], who noted a relationship between increased porosity and the enhancement of thermal and mechanical properties in bio-based composites. Therefore, further testing is needed to confirm these findings and assess the material’s potential for industrial production and, in general, for sustainable development and circular economy.

## 5. Conclusions

This study demonstrates the author’s method for creating an insulating material using agricultural biomass with fungal mycelium as a binder and thoroughly analyzed for thermal conductivity, density characteristics, and their qualitative relationship.

1. Research on thermal conductivity. In particular, the thermal conductivity coefficient measurements indicated quite close results across three lignocellulosic material variations and lie between 0.435 and 0.585 W·m^−1^·K^−1^. It is evident that the value remains consistent regardless of the type of agricultural residues.
2. Analysis by thermal conductivity—weight ratio (TC-WR) coefficient. During the investigation, the suggestion was made to utilize a qualitative TC-WR coefficient in evaluating thermal insulation materials, considering the inverse correlation between the density and thermal conductivity derivatives. The flax- and hemp-based samples displayed quite a close TC-WR meanings of 0.127 W^−1^·kg^−1^·m^4^·K and 0.123 W^−1^·kg^−1^·m^4^·K, respectively. However, the wheat-based sample showed a higher value of 0.148 W^−1^·kg^−1^·m^4^·K, which can be attributed to its lower density. In particular, the biomass with fungal mycelium binder demonstrated excellent values for this ratio and was one of the leading among biodegradable conventional materials included in the comparison. There were three that performed better: sheep wool (0.93 W^−1^·kg^−1^·m^4^·K), cellulose fibers (0.466 W^−1^·kg^−1^·m^4^·K), and mineral wool (0.197 W^−1^·kg^−1^·m^4^·K). Cork showed a middle performance among cellulosic agro ‘waste’, with mycelium having the indicative ratio of 0.139 W^−1^·kg^−1^·m^4^·K appropriately. Among the known analog composites derived from biomass and mycelium, suggested composites ranked third, fifth, and sixth out of ten types based on the TC-WR indicator (Table 3). Two first mycelium containing compounds in ranking may be attributed to the lower density of samples.
3. Predicting an optimal density. Our analysis aimed at identifying the optimal density to achieve the best proposed TC-WR coefficient. The values obtained exhibit a strong correlation with the findings from other comparable studies, although they show minor differences in absolute terms. For instance, Elsacker et al (2019) reported densities of 99 kg·m^−3^ for hemp biomass with mycelium, whereas our findings demonstrated the optimum density for this biomass at 85 kg·m^−3^. Specifically, a polynomial trend line was utilized to determine the most optimal production densities for wheat, hemp, and flax biomass, resulting in recommendations of 60–85–105 kg·m^−3^, respectively, to achieve the optimal TC-WR value for them. These densities strike a balance between thermal conductivity and weight of the insulating material, leading to TC-WR coefficients of 0.28–0.20–0.165 W^−1^·kg^−1^·m^4^·K for wheat–hemp–flax composites, respectively, which has a better potential for wheat with mycelium *Ganoderma lucidum* against mineral wool insulation with 0.197 W^−1^·kg^−1^·m^4^·K on average and wheat biomass with mycelium *Trametes versicolor* at 0.252 W^−1^·kg^−1^·m^4^·K.

## Figures and Tables

**Table 1:** Average component content of some perspective lignocellulosic biomass.

Type of Mass	Cellulose [%]	Hemicellulose [%]	Ash [%]	Lignin [%]	Source
Wheat	28–39	20–30	8–9	15	[20,21,22,23]
Hemp	70–76	11–17	3–4	2–5	[24]
Flax	73–76	12–16	3–4	2–5	[25]

**Table 2:** Measured values of Thermal conductivity [W·m^−1^·K^−1^] and Density [kg·m^−3^] for 15 test samples at an ambient temperature of 20°.

Biocomposite, Sample No.	Mass Before Drying, [kg]	Mass After Drying, [kg]	Initial Moisture Content MC [%]	Thermal Conductivity, *λ* [W·m^−1^·K^−1^]	Average Thermal Conductivity, *λ_Avg_* [W·m^−1^·K^−1^], dev. ± 0.9%	Density, *ρ* [kg·m^−3^]	Average Density, *ρ_Avg_* [kg·m^−3^], dev. ± 1 %
Flax + Mycelium, 1			59.60	0.045		158	
Flax + Mycelium, 2				0.046		220	
Flax + Mycelium, 3	0.160	0.064		0.047	0.046	146	170
Flax + Mycelium, 4				0.045		155	
Flax + Mycelium, 5				0.045		169	
Hemp + Mycelium, 1			53.08	0.047		227	
Hemp + Mycelium, 2				0.045		156	
Hemp + Mycelium, 3	0.152	0.071		0.046	0.047	119	172
Hemp + Mycelium, 4				0.046		138	
Hemp + Mycelium, 5				0.050		219	
Wheat + Mycelium, 1			54.82	0.043		137	
Wheat + Mycelium, 2				0.043		107	
Wheat + Mycelium, 3	0.135	0.061		0.056	0.048	142	138
Wheat + Mycelium, 4				0.054		156	
Wheat + Mycelium, 5				0.045		150	

## Data Availability

Some pictures on test methodology and lab data will be available at Mendeley Data, V1, reserved DOI: 10.17632/2f38ktnt92.1.
